# Mechanisms Underlying Tumor Suppressive Properties of Melatonin

**DOI:** 10.3390/ijms19082205

**Published:** 2018-07-27

**Authors:** Stephen C. Bondy, Arezoo Campbell

**Affiliations:** 1 Center for Occupational and Environmental Health, Department of Medicine, University of California, Irvine, CA 92697, USA; 2 Department of Pharmaceutical Sciences, Western University of Health Sciences, Pomona, CA 91766, USA; acampbell@westernu.edu

**Keywords:** melatonin molecular mechanisms, cancer treatment, cancer prevention, carcinogenesis

## Abstract

There is considerable evidence that melatonin may be of use in the prevention and treatment of cancer. This manuscript will review some of the human, animal and cellular studies that provide evidence that melatonin has oncostatic properties. Confirmation that melatonin mitigates pathogenesis of cancer will be described from both direct study of its effects on carcinogenesis, and from indirect findings implicating disruption of the circadian cycle. A distinction is made between the role of melatonin in preventing the initiation of the tumorigenic pathway and the ability of melatonin to retard the progression of cancer. Melatonin appears to slow down the rate of advancement of established tumors and there is evidence that it constitutes a valuable complement to standard pharmacological and radiation treatment modalities. There are instances of the beneficial outcomes in cancer treatment which utilize a range of hormones and vitamins, melatonin being among the constituents of the mix. While these complex blends are empirically promising, they are only briefly mentioned here in view of the confounding influence of a multiplicity of agents studied simultaneously. The last section of this review examines the molecular mechanisms that potentially underlie the oncostatic effects of melatonin. Alterations in gene expression following activation of various transcription factors, are likely to be an important mediating event. These changes in gene activity not only relate to cancer but also to the aging process which underlies the onset of most tumors. In addition, epigenetic events such as modulation of histone acetylation and DNA methylation patterns throughout the lifespan of organisms need to be considered. The antioxidant and immunoregulatory roles of melatonin may also contribute to its cancer modulatory properties. Naturally, these mechanisms overlap and interact extensively. Nevertheless, in the interest of clarity and ease of reading, each is discussed as a separate topic section. The report ends with some general conclusions concerning the clinical value of melatonin which has been rather overlooked and understudied.

## 1. Introduction

Melatonin is a neurohormone derived from serotonin and produced by several areas including the pineal gland, the intestine and within mitochondria [1,2]. It is best known for its regulation of the circadian rhythm [3]. Many reports indicate that the neurohormone may have various functions depending on the lifecycle stage. The first section of this review focuses on the role of melatonin during different phases of the human lifespan.

## 2. Role of Melatonin during Human Life Stages

The maternal hormonal environment is important for proper fetal brain development and besides thyroid hormone and glucocorticoids, melatonin is likely to participate in this regulation [4]. Melatonin released by the maternal system can easily enter the fetal circulation through the placenta [5]. First trimester human placental tissue expressed transcripts for melatonin receptors [6] and both human and rat placenta contained much higher levels of the neurohormone compared to serum [7]. This relatively high melatonin level may have an important role in imprinting of the fetal clock as well as providing protection [8]. Administration of melatonin (10 mg/kg) to pregnant rats was able to protect the fetal brain against oxidative mitochondrial damage induced by hypoxanthine and xanthine oxidase [9], indicating that the neurohormone may have this function in fetal development.

During infancy, melatonin is necessary for the evolution of the sleep-wake cycle and a delayed peak of secretion has been associated with fragmented nocturnal sleep [10]. The importance of proper control in infancy and childhood is demonstrated by the Smith-Magenis Syndrome which is a rare genetic disorder where melatonin secretion is inverted. Sleep disturbance is seen in all cases of children with the syndrome who also display a unique craniofacial feature accompanied by mental retardation [11]. Although other factors contribute to the neurological impairments, the reversal of melatonin secretion is evident and probably contributes to the clinical manifestations. It has been suggested that disturbance in early life sleep pattern formation may have long-term consequences in social behavior [12] and that the neurohormone may be useful as a co-treatment option for patients with mental disorders such as depression, schizophrenia, and bipolar syndrome [13].

Circadian rhythm disorders are most apparent in adolescents who commonly display difficulty falling sleep at night and have an inability to rise in the morning [14]. Dim light melatonin onset, an important component of the endogenous circadian phase, is delayed in adolescents [15]. Different profiles of melatonin release are compounded by changes during puberty that have environmental and psychosocial roots, leading this age group to tend to stay up late [16]. Such differences may partially underlie the difficulty in mood regulation, sleepiness, and learning problems in adolescents [17]. Since brain development is continuing during puberty, and melatonin modulates neuronal plasticity, the changes in this neurohormone (together with others such as sex and stress hormones) may incline this age group to many psychological disturbances [18].

During adulthood, melatonin may play a myriad of different functions. In young adults (ages 19–33), a short nap improved reward learning and this correlated with the magnitude of the melatonin response in the participants [19]. The ‘free radical theory of aging’ postulates that the accumulation of damage by oxidative mediators throughout an organism’s lifespan brings about the aging phenomenon [20]. During the adult phase of the human lifespan, melatonin appears to play a protective role as both an antioxidant and an immunomodulatory molecule [21]. As organisms age, the levels of melatonin decline sharply. In parallel, the number of chronic ailments rise. Many age-related disorders such as cardiovascular disease, cancer, and neurodegeneration have been associated with a decline in melatonin levels [22,23,24]. Thus, supplementation with melatonin may retard the onset of many such disorders.

## 3. Evidence That Melatonin Inhibits Cancer Onset

### 3.1. Direct In Vivo Evidence

There are many animal studies that point to the ability of melatonin to function as an oncostatic molecule. For instance, a study investigated the effect of melatonin on tumor incidence in aged B6C3F1 and CB6F1 mice. These are long-lived strains, not especially prone to cancer. At 15 months of age, less than 2% of animals have discernable tumors. However, at the last stage of their senescence, when the animals are over 26 months old, the incidence of visible tumors rises to 44%. If these animals were fed 40 ppm melatonin in their diet for the last 12 weeks of their life, the rate of tumor occurrence was cut by over half, to 18% [25]. Results from these long-lived mouse strains, with a sharp rise in basal tumor incidence at late stages of aging, are likely to be relevant to the human situation.

The above finding of a major reduction in incidence of spontaneous tumors in aged animals are complemented by reports involving use of chemically-induced or implanted cancers. Numerous reports show that melatonin can inhibit carcinogenesis in different tissues including breast, prostate, ovaries, liver, kidney, lung, pancreas, colorectum, skin, and the gastrointestinal system [26]. The effect of the neurohormone in breast cancer has been most studied [27]. In a rat model of 7,12-dimethylbenz(α)-anthracene (DMBA) induced mammary cancer, injection of 2.5 mg/kg of melatonin reduced tumor incidence from 79% to 22%. If the pineal gland was surgically removed, melatonin could only partially protect the animals [28]. The importance of the pineal gland is demonstrated in another study that evaluated prolonged melatonin administration of 0.5 mg/day/rat intraperitoneally for 93 days in rats exposed to constant light. A dramatic reduction (from 95% to 25%) in DMBA-induced mammary tumors was observed in intact rats. On the other hand, when pinealectomized rats were used, the tumor incidence in the melatonin group was only reduced to 53% compared to 83% in the control group [29]. Chronic use of the neurohormone in the drinking water reduced tumor incidence in a mammary cancer-prone mouse strain at the lower (2 mg/L) but not higher (20 mg/L) concentration [30]. This implies that melatonin concentration may have a biphasic effect on carcinogenesis.

Other forms of cancer can also be inhibited by melatonin. Intraperitoneal injection of 5 mg/kg melatonin for 20 weeks blocked *N*-nitrosodiethylamine (NDEA)-induced hepatic carcinoma in rats [31]. Furthermore, the growth rate of hepatic tumors implanted into rats is reduced by melatonin [32]. In a pancreatic cancer model, Syrian hamsters were treated with the carcinogenic compound *N*-nitrosobis (2-oxopropyl) amine (BOP). Melatonin was administered to the animals in drinking water (20 µg/mL) during tumor induction and postinduction periods. During both phases, the neurohormone was able to reduce the total number of cancerous nodules [33]. Melatonin suppresses the incidence of ovarian masses and adenocarcinomas following regional injection of 7,12-dimethyl-benz[a]anthracene (DMBA) in rats [34]. Overall, the onset of a large range of tumor types, induced in different animal models and by various carcinogens, is significantly inhibited by melatonin. The suppression of carcinogenesis in vivo is paralleled by reports of similar findings in cell culture.

### 3.2. Direct In Vitro Evidence

Few reports assess the effects of melatonin on normal cell transformation. However, there is some evidence that melatonin may inhibit the onset of carcinogenesis. Treatment with either 1 nM or 100 nM of melatonin significantly reduced the growth of MCF-7 human breast cancer cells and this was associated with the modulation of microRNAs (miRNAs) [35]. Genetic profiling of human breast cancer cells (MCF-7) showed that oncogenic genes (EGR3 and POU4F2) were downregulated while a tumor suppressor gene (GPC3) was upregulated after treatment with 1 nM melatonin [36]. In both androgen-dependent and independent prostate cancer cells, melatonin can stop the cell cycle progression and induced cellular differentiation [37]. In the hepatocarcinoma HepG2 cell line, melatonin administration caused growth inhibition that was associated with modulation of intracellular signaling pathways [38]. In aged individuals, who may be prone to cumulative DNA damage that may eventually lead to cellular transformations, the above processes may function in concert to inhibit initiation of carcinogenesis. However, the most critical evidence that link melatonin with a reduction in cancer incidence is provided by observations on the sleep-wake cycle described in the next section.

### 3.3. Indirect Evidence

The role of melatonin in the regulation of the sleep-wake cycle is well-known. The incidence of cancer increases with age. This is the same time that the production of melatonin in the pineal gland declines. It has been proposed that these two events may be interconnected [39]. Studies on nighttime workers who experience abnormal sleep-wake cycles prompted the international agency for research on cancer (IARC) to classify circadian rhythm-disrupting work as potentially carcinogenic [40,41]. The disruption of the circadian cycle by working at night or by the extended exposure to residential illumination caused by electric lighting are known to be disruptive to the diurnal flux of circulating levels of melatonin. Overall melatonin levels are depressed among nurses working at night [42,43]. Persistent night time illumination has been associated with excess incidence of breast cancer in females and prostate cancer in males and this has been associated with sex hormone production [44,45]. A meta-analysis collating reports from several sources of female night shift workers, found a convincing association with excess breast cancer, CI: 1.36–1.61 [46]. Reduced melatonin levels and endocrine effects induced by night shift work are likely to interact in a harmful manner [47]. Nightshift work has been found disruptive to components of the immune system that are influenced by the circadian cycle [48].

The epidemiological findings from human populations, which are subject to a range of potential confounders, are strengthened by laboratory studies under more controlled conditions involving animals subjected to a disrupted circadian cycle. The development of breast cancer in mice prone to this condition, is increased after chronically alternating light cycles [49]. The increased rates of tumor growth found in animals exposed to continuous light cannot be reversed by melatonin administration [50]. Continuous light leads to permanent depression of levels of circulating melatonin, the normal nighttime elevation being blocked by over 99% [51]. This illustrates the importance of maintaining intrinsic endogenous rhythmicity. Other studies involving circadian disruption, have documented that continuous exposure to light results in increased resistance of breast cancer to therapeutic drugs such as tamoxifen [52]. Disruption of the circadian cycle by exposure to dim light at night in mice bearing human breast cancer xenografts, leads to complete loss of tumor sensitivity to doxorubicin chemotherapy. In contrast, exogenous melatonin administered at night retarded breast cancer development [53].

## 4. Evidence That Melatonin Slows Rate of Cancer Progression and Metastasis

### 4.1. Animal Models

There are many reports of melatonin impeding both tumor growth and spread to other tissues. Melatonin reduced the size of tumors in female BALB/c nude mice that were subcutaneously injected with thyroid cancer cells [54]. Xenografts of hepatocellular carcinoma cells in a nude mouse model of cancer growth demonstrated substantial reduction of tumor size after chronic treatment with 40 mg/kg of melatonin. Thus, melatonin treatment can reduce the progression of different types of cancers by inhibiting uncontrolled cell proliferation. Melatonin has also been shown to hinder cancer metastasis. In female athymic nude mice, melatonin reduced lung metastases after intravenous injection of a human breast cancer cell line (MDA-MB-231) [55]. The development and peritoneal spread of tumors in male BALB/c nude mice inoculated intraperitoneally with gastric cancer cells was significantly suppressed by 5 mg/kg melatonin administered twice weekly [56]. The anti-metastatic action of melatonin at pharmacologically relevant concentrations have also been reported in a renal carcinoma non-obese diabetic SCID mouse model [57]. These reports support the concept that melatonin is exerting its oncostatic properties at all stages of carcinogenesis.

### 4.2. Cell-Based Models

There is an array of in vitro studies that use transformed cell lines and that demonstrate the capability of melatonin to inhibit proliferation and cell migration. These have been extensively listed in a recent review [26]. Therefore, we will limit this section to a few examples. In the central nervous system, glioma tumor growth is the most common malignancy with high invasiveness. Melatonin (1 mM) was able to inhibit glioma cell growth in both human tissue specimens and two different glioma cell lines [58]. A three-dimensional model of breast cancer was developed by growing human breast cancer cell line MCF-7 in stem cell media to form mammospheres. A 24 h treatment with melatonin (1 mM) reduced both the migration and invasiveness of MCF-7 mammospheres [59]. A similar inhibition of tumor cell migration by melatonin has been found in isolated systems such as adenocarcinoma cells [60]. Unfortunately, few reports directly compare the effects of melatonin in enhancing chemotherapeutic-induced cytotoxicity in transformed cells. However, there are a handful of published reports that indicate melatonin may have selective toxicity to cancer cells. Synergistic cytotoxicity of melatonin and anticancer drugs toward leukemia lymphocytes but not normal lymphocytes has been described [61]. Furthermore, viability of malignant cells but not their normal counterparts is reduced by melatonin [62]. SIRT1 is a member of the sirtuin family of proteins that plays a role in chromatin remodeling and gene expression. Although best understood as an anti-aging factor, it is also thought to regulate cancer development [63]. Melatonin treatment leads to elevated SIRT1 activity in isolated cells and in animal models. However, an opposite effect whereby melatonin inhibits SIRT1 is found in some tumor cells [64]. Such differential susceptibility of cancerous cells compared to normal healthy cells increases the therapeutic potential of melatonin and merits further investigation.

### 4.3. Clinical Trials

A systematic review of randomized controlled trials from 1992 to 2003 of the effectiveness of melatonin in solid tumor cancer patients (*n* = 643) showed that the neurohormone can reduce risk of death irrespective of dose or type of cancer [65]. Reports of increased survival time in patients suffering from untreatable metastatic solid tumors [66] and a better response to standard chemotherapeutic agents when co-administered with melatonin [67] reflect the findings from experimental animals. Most of the clinical trials that have been conducted with melatonin are based on adjunct therapy to relieve patients from adverse effects of other more aggressive therapeutic regimens. Co-administration of melatonin together with standard chemotherapeutic agents may alter survival rates and quality of life of patients. For example, patients that were treated with melatonin (20 mg/day) in conjunction with cisplatin and etoposide had a greater tumor regression as well as a better 5-year survival rate [68]. A meta-analysis of 21 carefully validated clinical trials, indicated that melatonin can benefit cancer patients who are also receiving chemotherapy, radiotherapy, supportive therapy, or palliative therapy by improving survival and ameliorating the side effects of chemotherapy [69]. There have been individual case reports of complete remission obtained in patients with several immune modulators including melatonin [70]. In addition, the amount of morphine taken for pain in cancer patients may be reduced [71]. However, there are also reports of melatonin’s ineffectiveness in altering the extent of fatigue found in patients with advanced cancer [72]. This is perhaps unsurprising since melatonin can cause sleepiness. The time of administration of melatonin in the circadian cycle, is likely to be critical in determining its clinical effects and failure to take this into account may partially explain the lack of consistent findings in this area. Overall, there is a paucity of reliable reports in this area, perhaps because of the expense of clinical trials and the lack of a commercial incentive to develop the utility of a non-proprietary agent such as melatonin. However, considering how nontoxic the neurohormone is in context to the very toxic chemotherapeutic agents currently in use in cancer patients, there is a critical need for large well-conducted clinical trials to better explore the benefits of melatonin [73].

### 4.4. Melatonin as A Co-Therapeutic Agent

There are instances of the beneficial outcomes in cancer treatment which utilize a range of hormones and vitamins, melatonin being among the constituents of the mix [74,75]. The role of melatonin in these mixtures is however difficult to ascertain.

## 5. Mechanisms

**Figure 1:**
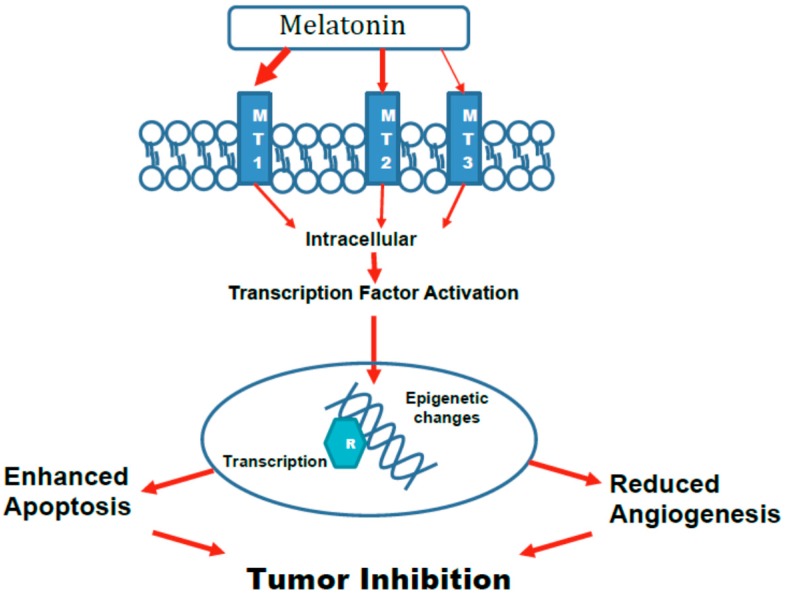
The effects of melatonin on carcinogenesis that directly Inhibit tumors. Melatonin interacts with its surface receptors to activate intracellular signaling cascades. The size of the arrows reflects the importance of the receptor subtype. Intracellular signaling leads to activation of transcription factors that cause changes to the DNA in a manner that enhance apoptosis of cancer/pre-cancer cells and reduce angiogenesis which is necessary for tumor growth and metastasis.

**Figure 2:**
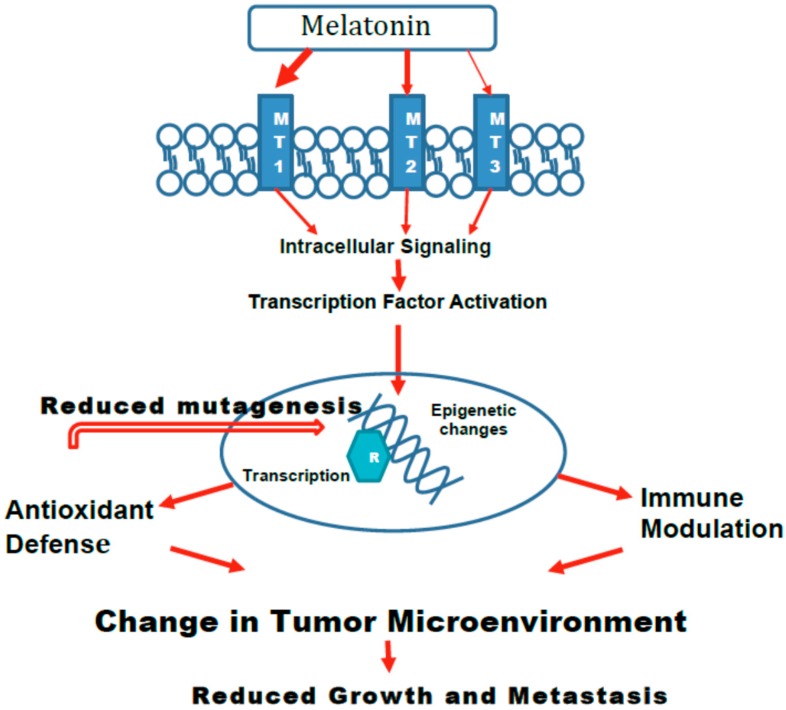
The effects of melatonin on carcinogenesis that Indirectly Inhibit tumors. The initial steps in the indirect action of melatonin on suppressing tumors are the same as Figure 1. Melatonin interacts primarily with MT1 receptors to activate intracellular signaling that leads to activation of transcription factors. The subsequent changes to the DNA provide up-regulation of antioxidant defenses that can reduce mutations that lead to initiation of cancer. Furthermore, the up-regulation of antioxidants and modifications to immune responses alter the microenvironment of cancer cells in a manner that reduces cancer progression and metastasis.

As previously discussed, endogenous melatonin appears to have a biological role throughout the lifespan of an individual. Beginning from fetal transport of the neurohormone from maternal circulation, to the continuous production throughout the lifespan, and finally to the decline in the aging organism, melatonin appears to orchestrate beneficial physiological changes necessary for adaptation. Thus, it is not surprising that this chemical, which was initially understood as a mere circadian rhythm adjustor, has now evolved to a molecule with pleiotropic properties [3]. As such, the tumor suppressive function of melatonin encompasses several overlapping mechanisms. These may directly impede the formation and growth of tumors (Figure 1) or indirectly effect cancer cell growth or metastasis by changing the microenvironment (Figure 2).

### 5.1. Epigenetic Changes

The study of changes in gene expression, such as methylation patterns and histone modifications, which are not coded in the DNA sequence, are referred to as epigenetics [76]. The epigenetic control of tumor suppressor genes and cellular proliferation factors play a role in carcinogenesis and accumulating evidence indicate that melatonin may influence initiation, proliferative, and metastatic phases of cancer through modulating of epigenetic mechanisms [77].

#### 5.1.1. Histone Acetylation

By enabling a decreased rate of histone acetylation at specific loci, melatonin can inhibit matrix metalloproteinase. This enzyme is elevated in several tumor species and permits movement between cells, increasing their motility and facilitating metastasis [78]. Melatonin can also induce hyperacetylation at other sites on the chromatin, resulting in suppression of tumor cell proliferation and apoptosis [79]. This effect probably involves activation of the MT1 receptor [80]. Melatonin may have different effects on various isoforms of histone deacetylase, all of which diminish tumor progression.

#### 5.1.2. Histone and DNA Methylation

Aberrant activity of histone lysine-specific demethylase is associated with many tumors. Excess levels of this enzyme lead to decreased methylation at histone 3 lysine 4, and this leads to reduced expression of tumor suppressor genes. The containment of proliferation of oral squamous cell carcinoma by melatonin has been related to its inhibition of this enzyme [81]. The means by which melatonin can block the tumor-promoting effect of exposure of animals to continuous light is likely to involve widespread methylation of DNA [82]. Such inhibition has been ascribed to specific down-regulation of the adenosine triphosphate-binding cassette transporter ABCG2/BCRP [83].

#### 5.1.3. miRNA

A means by which melatonin inhibits tumor cell proliferation and migration is by decreasing miR-24 levels. miR-24 is upregulated in several types of tumor. This micro RNA inhibits several genes associated with DNA repair while promoting those relating to cell division. Melatonin, by way of the MT1 or MT2 receptor brings about a decrease in miR-24 levels [84]. Many other examples of melatonin acting to reduce tumor progression by way of alteration of miRNA expression, have been described. These include both up-regulation [85,86] and down-regulation [87] of specific miRNAs. A considerable number of miRNAs are differentially expressed in melatonin-treated tumor cells [35].

### 5.2. Melatonin-Induced Effects on Age-Related Changes in Gene Expression and Immune Modulation

The inflammatory component of natural immunity protects an organism from a variety of pathogenic and endogenous stressors. However, as an individual ages, the immune system loses its ability to respond properly. This process is referred to as “immunosenescence” and underlies diminished protection offered by vaccines and inability to effectively regenerate damaged tissue [88,89]. The overall profile of gene expression is altered with aging. One characteristic is a gradual increase in the basal activity of levels of genes relating to immune function. The activity of pro-inflammatory genes is elevated even in the absence of an exogenous provocative stimulus [90]. Extended treatment of aged animals with melatonin has been found to reverse this change and to restore the gene expression pattern to a form more closely resembling that of younger animals [90]. Since cancer incidence rises sharply with aging [91], such reduction of indices of inflammation by melatonin in part account for the lower incidence of tumors found in senescent animals treated with melatonin. A concomitant of any deceleration of the events associated with aging is likely to include a reduction of overall cancer incidence. It is likely that many of the protective properties of melatonin can be attributed to reversal of age-related modifications of profiles in gene expression [92].

Treatment of senescent animals with the inflammation-provoking lipopolysaccharide (LPS), leads to greater expression of chemokine S100-A8, but to down-regulation of expression of chemokine Cxcl1. Pretreatment with melatonin caused the expression of either cytokine to LPS in older animals, to return to that of young animals [93]. Thus, melatonin either inhibited or enlarged the inflammatory response of aged animals, but the change effected was invariably in a direction resembling the corresponding reaction of young animals. Melatonin should thus be considered as a subtle modulator rather than an unselective repressor, of immune-based inflammatory processes.

### 5.3. Activation of Transcription Factors Leading to Altered Gene Expression and Apoptosis

The ability of melatonin to selectively lead to apoptosis in tumor cells is likely to encompass more than one underlying mechanism. Melatonin can cause apoptotic events in tumor cells at a rate many fold greater than in non-tumor cells. This important disparity has been attributed to different effects of melatonin on calcium transport systems [94]. Melatonin facilitates dephosphorylation and nuclear import of histone deacetylase 4, leading to inactivation of calmodulin-dependent protein kinase II alpha and apoptosis [79]. Other means by which melatonin promotes apoptosis in tumor cells include selective blockade of activating transcription factor 6 leading to down-regulation of cyclooxygenase-2 (COX-2) expression [95]. Other influence of melatonin on transcription factors that lead to apoptosis, include the JNK/c-jun pathway and activation of caspase-3 [96]. Melatonin seems to induce apoptotic events in cancer cells by concurrent suppression of multiple signaling pathways [97]. Another important transcription factor activated by melatonin is Nrf2 which derepresses a wide range of antioxidant and anti-inflammatory genes. Activity of this factor is associated with decreased progression of colitis-associated colon carcinogenesis in a mouse model [98]. The initiation of the Nrf2 sequence is brought about by its phosphorylation and nuclear translocation. Melatonin may further this process by elevation of intracellular Ca and consequent activation of PKC [99]. The ability of melatonin to ameliorate progression of mouse model of colitis-associated carcinogenesis in a mouse model, is accompanied by increased expression of Nrf2 and the antioxidant enzymes associated with this activation [98].

Many of the attributes of melatonin have been associated to its properties as an antioxidant. Such functions are unlikely to comprise major free radical scavenging by the molecule itself. The intracellular concentration of melatonin is very low (picomolar amounts) [100] when compared to levels of water-soluble antioxidants such as glutathione which is present in millimolar amounts and lipid soluble α-tocopherol present in cells at around 5 µM [101]. In the duck pineal gland, the maximum melatonin concentration reached during the circadian cycle is only 16 nM [102]. Melatonin content is higher in membranes than in cytosol [51]. This is most likely due to its relatively greater lipophilic nature, but the concentrations in mitochondrial and other membranes still do not exceed around 80 nM. Most of the reports of melatonin acting by means not involving its receptors [103] are based on the use of much larger concentrations in experimental systems than those found in intact animals. The pronounced antioxidant and anti-inflammatory activities of melatonin that are undoubtedly important determinants of its oncostatic role, are probably largely mediated by its activation of receptors and resulting effects on a wide range of transcription factors and signaling pathways. However, large doses of melatonin are used in some clinical studies and in such instances, melatonin may reach concentrations where it has a significant direct antioxidant effect.

### 5.4. Angiogenesis

Expansion of angiogenesis is a major feature of tumor progression, and its inhibition is an important means by which melatonin retards the spread of tumors. The mechanisms underlying this are manifold but ultimately lead to a reduction of expression levels of genes required for angiogenesis [104]. Inhibition of expression of vascular endothelial growth factor is a central part of this targeting [105]. Melatonin-promoted inhibition of inflammatory events also plays a role in retarding blood vessel proliferation [106] and melatonin can simultaneously reduce content of angiogenic and inflammatory proteins in a mammary tumor cell line [62]. In hepatocellular carcinoma cells, prazosin, an MT3 receptor antagonist, can strongly upregulate the IL-6 gene, involved in inflammatory events and in promoting angiogenesis [107].

### 5.5. Role of Various Melatonin Receptors

The triggering of transcription factors and signaling pathways by melatonin largely entails the activation of specific melatonin receptors. All the known melatonin receptors have at one time or another been implicated in furthering the cancer-retarding properties of melatonin.

#### 5.5.1. MT1 and MT2 Receptors

Receptors MT1 and MT2 are similar and not readily distinguished by pharmacological mimetics. Ramelteon is an agonist of both these receptors and inhibits the proliferation and invasiveness of an estrogen receptor-positive endometrial cancer cell line. This effect is blocked by the MT1/MT2 receptor antagonist luzindole. This results in inhibition of expression of MMP-2 and MMP-9 genes which regulate metalloproteinase activity [108]. Similar findings have been reported for oral cancer cell lines [78]. This family of zinc dependent endopeptidases degrade the extracellular matrix and can thus allow tumor cells to migrate, invade and spread to form metastases [109]. Use of more selective inhibitors suggests that the MT1 rather than the MT2 receptor is more effective in suppression of a mouse colon cancer cell line [110] and in suppression of oral carcinogenesis [111].

Other direct evidence for the role of MT1 specifically, in suppression of mammary tumors, is that melatonin is more effective in decreasing tumor viability in cells overexpressing MT1 [112]. In breast cancer cells overexpressing the MT1 gene, the expression of the estradiol-induced genes was more strongly reduced by treatment with melatonin than in the parental cells [113]. The inhibition of growth of glioma cell lines by melatonin is also proportional to the expression level of MT1 in glioma [58]. MT1 receptors in the uterus decline with age, and this is reflected by reduced sensitivity of tumors to exogenous melatonin in aged rats [114].

A recent report on the mitochondrial synthesis on melatonin reveals that, within this organelle, MT1 receptors respond to melatonin by activating G proteins located in the intermembrane space and thereby inhibit stress-mediated cytochrome *c* release [115]. This has important implications for the prevention of neurodegeneration associated with mitochondrial cytochrome *c* release and downstream caspase activation.

#### 5.5.2. MT3 (Quinone Oxidoreductase) Melatonin Binding Site

The MT3 receptor binding site has enzymic activity as quinone reductase 2. There is also evidence suggesting a role for the MT3 receptor/binding site in oncostatic effects of melatonin. The neurohormone reduces the viability of human colorectal cancer HT-29 cells and cervical cancer HeLa cells. While blockade of MT1 and MT2 receptors with luzindole had no effect on this reduction, prazosin, an MT3 antagonist [107] prevented the cytotoxic effect of melatonin. In this case, the ability of melatonin to selectively cause cytotoxicity and promote apoptosis in tumor cells appears to be mediated by activation of the MT3 site receptor [116]. This remains controversial and the exact nature and role of this receptor/enzyme needs further investigation and clarification. It should be borne in mind that prozosin is a multi-target drug, and thus interpretation of its effect on any cellular system should be taken with extreme caution.

#### 5.5.3. RZR/RORα: Retinoid-Related Orphan Nuclear Hormone Receptor

This group of receptors may allow melatonin to bind to transcription factors. However, the question of whether melatonin interacts directly with these nuclear receptors has been a source of controversy [117]. RORα promotes apoptosis and is reduced in human gastric cancer [118]. Studies with knockout mouse lacking the RORα show that this factor is important in enhancing the anti-inflammatory [119] and the anti-proliferative [120] effects of melatonin. The mechanism of inhibition of proliferation of human gastric cancer cells by melatonin also involves the down-regulation of expression of the RORγ receptor which is over-expressed in many tumor types [121]. However complete knockout of the gene in adult mice leads to 50% of the knockout mice developing lymphoma [122]. Thus, the precise degree of activity of this receptor, is critical in determining whether it can be harmful or protective. As is the case with the MT3 receptor, this is an area where many uncertainities persist, and the physiological function of this orphan nuclear hormone receptor remains unresolved.

## 6. Conclusions

Melatonin cannot be described as a simple indiscriminate antioxidant or anti-inflammatory chemical. The complicated nature of melatonin’s properties is illustrated by the fact that, in regular cells, melatonin elevates expression of the anti-apoptotic gene, Bcl-2 [123] while in transformed cells; expression of Bcl-2 is diminished thereby promoting apoptosis [124]. The means by which melatonin exerts its protective effects are diverse. There is no one event underlying all the metabolic consequences caused by this neurohormone. It is probable that the general sequence of steps in enabling melatonin to exert its characteristic influences, initially involves its binding to several of its known receptors. While a wide range of consequences arises from this, responses following receptor stimulation broadly involve signaling pathways mediated by transcription factors. The eventual outcome of this impinge not merely directly on gene expression but also on epigenetic events that further regulate the initiation and progression of carcinogenic pathways.

There is a large body of evidence showing that the progression of an already established tumor can be attenuated but not totally arrested by melatonin. In this circumstance, melatonin is best thought of simply as an adjuvant to conventional treatments. However, there is a strong argument to be made for the ability of melatonin to prevent the onset of critical steps of cancer initiation and thus block commencement of tumors. This may be especially true in middle-aged individuals who may not have as yet accumulated sufficient amount of transformations necessary for carcinogenesis. Thus, melatonin modulation of epigenetic factors may be most beneficial in this population of individuals. The possibility then arises that melatonin may be able to effect a significant reduction of overall cancer incidence if used at the right dose and at a proper stage of human development and aging. The very low toxicity of melatonin together with its low cost and ready availability make it a good candidate for widespread usage as a potential preventive measure against cancer.
